# Genome Size Variation and Evolution Driven by Transposable Elements in the Genus *Oryza*

**DOI:** 10.3389/fpls.2022.921937

**Published:** 2022-07-07

**Authors:** Shuang-feng Dai, Xun-ge Zhu, Ge-rang Hutang, Jia-yue Li, Jia-qi Tian, Xian-hui Jiang, Dan Zhang, Li-zhi Gao

**Affiliations:** ^1^Institution of Genomics and Bioinformatics, South China Agricultural University, Guangzhou, China; ^2^Plant Germplasm and Genomics Center, Kunming Institute of Botany, Chinese Academy of Sciences, Kunming, China; ^3^College of Tropical Crops, Hainan University, Haikou, China

**Keywords:** *Oryza*, genome size, flow cytometry, *k*-mer analysis, transposable elements

## Abstract

Genome size variation and evolutionary forces behind have been long pursued in flowering plants. The genus *Oryza*, consisting of approximately 25 wild species and two cultivated rice, harbors eleven extant genome types, six of which are diploid (AA, BB, CC, EE, FF, and GG) and five of which are tetraploid (BBCC, CCDD, HHJJ, HHKK, and KKLL). To obtain the most comprehensive knowledge of genome size variation in the genus *Oryza*, we performed flow cytometry experiments and estimated genome sizes of 166 accessions belonging to 16 non-AA genome *Oryza* species. *k*-mer analyses were followed to verify the experimental results of the two accessions for each species. Our results showed that genome sizes largely varied fourfold in the genus *Oryza*, ranging from 279 Mb in *Oryza brachyantha* (FF) to 1,203 Mb in *Oryza ridleyi* (HHJJ). There was a 2-fold variation (ranging from 570 to 1,203 Mb) in genome size among the tetraploid species, while the diploid species had 3-fold variation, ranging from 279 Mb in *Oryza brachyantha* (FF) to 905 Mb in *Oryza australiensis* (EE). The genome sizes of the tetraploid species were not always two times larger than those of the diploid species, and some diploid species even had larger genome sizes than those of tetraploids. Nevertheless, we found that genome sizes of newly formed allotetraploids (BBCC-) were almost equal to totaling genome sizes of their parental progenitors. Our results showed that the species belonging to the same genome types had similar genome sizes, while genome sizes exhibited a gradually decreased trend during the evolutionary process in the clade with AA, BB, CC, and EE genome types. Comparative genomic analyses further showed that the species with different rice genome types may had experienced dissimilar amplification histories of retrotransposons, resulting in remarkably different genome sizes. On the other hand, the closely related rice species may have experienced similar amplification history. We observed that the contents of transposable elements, long terminal repeats (LTR) retrotransposons, and particularly LTR/*Gypsy* retrotransposons varied largely but were significantly correlated with genome sizes. Therefore, this study demonstrated that LTR retrotransposons act as an active driver of genome size variation in the genus *Oryza*.

## Introduction

Genome size refers to the DNA amount of an unreplicated gametic nuclear, and it is one of the most important characteristics of organisms (Swift, 1950; Soltis et al., 2003; Greilhuber et al., 2005). The latest release of The Kew Plant DNA C-value database^1^ has collected a C-value of 10,770 angiosperm species, which varied remarkably by more than 2,100-fold, ranging from 0.07 pg/1C in *Genlisea aurea* to 152.23 pg/1C in *Paris japonica*. The genome size within the same genus usually varies greatly, even different individuals within the same species may have variable genome sizes (Gregory, 2005; Tsutsui et al., 2008; Huang et al., 2013). Genome size not only reflects the biological adaptability to a certain extent (Sun et al., 2018; Piégu et al., 2020) but also plays an important role in the phylogeny and species classification (Bures et al., 2004; Huang et al., 2013). With rapid progress in genome sequencing technology and the reduction of sequencing costs, more and more species’ nuclear genomes will be completed. It is broadly recognized that, for the species to be sequenced, the genome size can provide a reference for the amount of data required for whole-genome sequencing and evaluate the integrity of genome assembly results.

Genome size can be measured either in picograms (pg) or million base pairs (Mbp, 1 pg = 978 Mbp) (Dolezel et al., 2003), corresponding to different methods to estimate genome sizes. It is widely accepted that the flow cytometry analysis serves as the main method to estimate the nuclear DNA contents (Bennett and Leitch, 2005; Dolezel and Bartos, 2005), which calculates the relative DNA content of each nucleus by quantifying the fluorescence emitted by each stained nucleus (Dolezel and Bartos, 2005). The estimation of genome size by flow cytometry analysis requires a species with known genome size as an external or internal standard. When using external standards, the factors like the random drift error of the instrument, the influence of secondary metabolites in target species, and standard species on the binding of dye and DNA will lead to inevitable experimental errors. These can nevertheless be avoided by using an internal standard, in which the nuclei of target species and internal standard species are isolated, stained, and analyzed simultaneously (Price et al., 2000; Dolezel and Bartos, 2005; Bennett et al., 2008). An ideal DNA reference standard should have a genome size close to the target species, which can avoid the risk of non-linearity and offset errors (Bagwell et al., 1989; Dolezel and Bartos, 2005). On the other hand, peaks of the two species with fairly close genome sizes could overlap when using an internal standard, making it impossible to accurately estimate the genome size. Therefore, selecting the appropriate internal standard depends on the genome size of different species, which is crucial to guarantee the accurate results of flow cytometry analysis.

Alternatively, the genome size can be estimated by bioinformatics-based *k*-mer analyses of Illumina sequencing data (Li and Waterman, 2003; Liu et al., 2013; Sun et al., 2018; Mgwatyu et al., 2020), which is independent of a species with known genome size as an internal or external standard like flow cytometry. Therefore, *k*-mer based method should be theoretically more accurate than flow cytometry analysis in estimating the absolute genome size of species. Consequently, more and more researchers tend to use both flow cytometry and *k*-mer-based methods to verify and then compare each other in order to obtain a more accurate genome size (Guo et al., 2015; He et al., 2016; Mgwatyu et al., 2020; Pflug et al., 2020). Many *k*-mer-based genome size estimation methods have been published in recent years. Sun et al. (2018) compared six *k*-mer-based methods for estimating genome size and found that gce (Liu et al., 2013) still performed well under the conditions of low base coverage, high heterozygosity level, and high sequencing error rate.

As one of the most important genera of Gramineae, *Oryza* species not only provide staple food for half of the world’s population but is also an important study model for the plant research community. It is commonly recognized that there are about 27 species in the genus *Oryza* nowadays (Stein et al., 2018; Wing et al., 2018), including 11 genome types, of which six are diploid (AA, BB, CC, EE, FF, and GG) and five are tetraploid (BBCC, CCDD, HHJJ, HHKK, and KKLL). Rice breeders and geneticists have long focused on the AA genome type species in the past decades, among which cultivated rice is included because a high-quality reference genome for each AA genome *Oryza* species has been obtained (Goff et al., 2002; Yu et al., 2002; Huang et al., 2012; Reuscher et al., 2018; Stein et al., 2018; Wu et al., 2018; Li et al., 2020a; Xie et al., 2021). However, in the genus *Oryza*, a large number of non-AA genome species have not been sequenced except for *Oryza brachyantha* (Chen et al., 2013), *Oryza granulata* (Wu et al., 2018; Shi et al., 2020), *Oryza coarctata* (Mondal et al., 2017, 2018; Bansal et al., 2021), *Oryza punctata* (Stein et al., 2018), *Oryza alta* (Yu et al., 2021), and the three CC genome species, *Oryza officinalis, Oryza eichingeri*, and *Oryza rhizomatis* (Shenton et al., 2020). It is well known that an accurate estimation of genome size is essential for genomics research since it is related to genome assembly difficulties and costs. Although continuous efforts have been put into estimating genome sizes of *Oryza* species for decades, for some rice species, genome sizes measured by different laboratories were not consistent with each other probably as a result of the innovation of equipment and technology, regeneration of nuclear isolation buffer in flow cytometry experiments, genome optimization of internal reference species and the conversion factor for picograms to base pairs (Dolezel et al., 2003), and different selection of internal standard as well. For instance, the 2C-value of *O. rhizomatis* (IRGC103410) measured by Miyabayashi et al. (2007) was 1.92 ± 0.17 pg, namely about 926 Mb, but Shenton et al. (2020) reported that the genome size of *O. rhizomatis* (IRGC103410) was approximately 559 Mb after sequencing and assembling the *O. rhizomatis* genome (IRGC103410). Another example is that the 2C-value of *O. ridleyi* (IRGC100821) measured by Miyabayashi et al. (2007) was 2.03 ± 0.33 pg, while the 2C-value of *O. ridleyi* (IRGC100821) was 2.66 ± 0.14 pg (Ammiraju et al., 2006), giving 31% differences between these two studies. Given the importance of genome size as a metric for genome characterization, it is necessary to accurately estimate genome sizes of *Oryza* species for the exploration of wild rice germplasm resources.

It has been proven that retrotransposons, especially LTR retrotransposons (LTR-RTs), play an important role in plant genome size variation (Gao et al., 2004; Havecker et al., 2004; Ma et al., 2004; Wicker and Keller, 2007; Zhang and Gao, 2017; Zhou et al., 2021). LTR-RTs belong to Class I transposons, which act by a “copy-paste” mechanism to result in the increase in genome size. An intact LTR-RTs includes two long terminal repeats (LTR) flanking elements that usually start with 5’TG-3’ and end with 5’-CA3’. The internal sequence between two LTRs consists of two genes: *GAG* and *POL*. The *GAG* gene encodes structural protein for virus-like particles, while the *POL* gene encodes four proteins domains, including a protease (PR), a ribonuclease H (RH), a reverse transcriptase (RT), and an integrase (INT), the relative order of RT and INT was used to classify the LTR-RTs family into *Copia* (PR-INT-RT) and *Gypsy* (PR-RT-INT) superfamily in the plant, which can further divide into an enormous number of lineages (Wicker et al., 2007). The proportions of LTR-RTs in the diploid *Oryza* genomes largely varied from approximately 7.51–61.98% in previous studies (Reuscher et al., 2018; Stein et al., 2018; Wu et al., 2018; Li et al., 2020a,b; Xie et al., 2021). Since the genome assembly quality may affect the repeat annotation, the availability of high-quality rice reference genomes provides an unprecedented opportunity to understand how transposable elements drive genome size variation and evolution in the genus *Oryza*.

In this study, we performed flow cytometry experiments to accurately estimate genome sizes of 166 accessions from 16 non-AA genome *Oryza* species. We then generated 10× depth Illumina sequencing short reads of two accessions for each species to calculate genome sizes by the *k*-mer-based method. The relatively accurate genome sizes for each species obtained by combining results of flow cytometry and *k*-mer analyses have comprehensively updated the genome size dataset of the genus. We researched patterns of genome size variation in the context of the *Oryza* phylogeny reconstructed based on SNPs located on fourfold-degenerate sites and examined the contribution of LTR-RTs to genome size variation, which further our understanding of genome size evolution in the genus *Oryza*.

## Materials and Methods

### Plant Material

Rice materials used in this study (Supplementary Table 1) were kindly provided by International Rice Research Institute (IRRI) and cultivated in the greenhouse of South China Agricultural University (Guangzhou). Fresh leaves taken from plants were immediately wrapped with filter paper soaked in sterile water and put into a 4°C refrigerators until performing flow cytometry in 2 h.

### Flow Cytometry Analysis

About 7 mg leaves were collected from the fresh plants to be tested and the internal standard plants and then were ground together in a centrifuge tube containing 1 ml of lysate buffer (0.1 M citric acid, 0.5% Triton X-100 in distilled water) to prepare the nuclear suspension (Hanson et al., 2005). Samples were ground at a frequency of 25 Hz for 48 s in a 400 MM TissueLyser (Retsch, Mettmann, Germany). The ground homogenate was filtered through a 30 μm filter into a new 2 ml centrifuge tube. The filtrate was treated with 50 μl RNase (3 mg/L) and incubated at 37°C for 30 min to remove RNA inside. *O. sativa* L. ssp. *japonica* cv. *Nipponbare* (0.7955 pg/2C, 389 Mb/1C) and *O. granulata* (1.5812 pg/2C, 773 Mb/1C) were used as internal standards (Sasaki, 2005). A total of 0.4 ml of treated filtrate was added to 2 ml of the PI staining solution, which comprised 11.36 g of Na_2_HPO_4_, 12 mL of PI stock (1 mg/ml), and 20 ml of 10× stock (100 mM sodium citrate, 250 mM sodium sulfate) made up to 200 ml with double-distilled water. The mixture was then fully mixed and incubated at room temperature (20–25°C) for 20 min in the dark. The stained samples were analyzed on a Sysmex CyFlow Ploidy Analyser (Sysmex Partec, Germany) with an argon laser light source (532 nm wavelength). Samples were run at a constant flow rate (0.4 μl/s) until at least 10,000 nuclear were collected for each sample. The experimental data were further analyzed by FCS Express V3 flow cytometry software and gated to selectively visualize all cells of interest which gather densely in a dot plot map while eliminating results from unwanted particles. The coefficient of variation (CV), which was equal to standard deviation/peak mean × 100%, was used to evaluate the credibility of the results. When the CV value of three replicates for each sample was less than 5%, the results were considered to be reliable. The absolute DNA amount of a sample was calculated based on the value of the G0/G1 peak means: [(sample G0/G1 peak mean)/(standard G0/G1 peak mean)] × standard 2C DNA content (pg). The formula, 1 picogram (pg) = 0.978 × 10^9^ base pair (bp), was used when converting picogram to the base pair (Dolezel et al., 2003).

### *k*-mer Analysis

To confirm the results of flow cytometry, we also estimated the genome size for each species by using *k*-mer analysis, which was successfully employed in some species such as insects, Rooibos, and *Bemisia tabaci* (Guo et al., 2015; He et al., 2016; Mgwatyu et al., 2020). Total DNA was extracted from leaf tissues by using a modified CTAB method (Porebski et al., 1997), and 150 bp paired-end reads were produced using the Illumina sequencing platform. Fastp (version.21) was used to control the quality of raw sequence data with parameters: -q 30 -u 40 -l 50 (Chen et al., 2018). High-quality clean reads were then used to estimate the rough genome size by using gce (gce-1.0.2, gce-alternative) with the *k*-mer size set to 17 (Liu et al., 2013; Sun et al., 2018).

### Phylogenetic Reconstruction

The raw Illumina sequencing data sources were listed in Supplementary Table 2. Adaptors and reads with more than 40% of the bases with low-quality bases (*Q* < 30) were trimmed from raw reads by fastp (fastp-0.21.0)(Chen et al., 2018). Clean reads longer than 50 bp were aligned to the *O. sativa* L. ssp. *japonica* cv. *Nipponbare* genome (IRGSP-1.0_genome) using BWA-MEM (version.7.17-r1188) with default parameters (Li and Durbin, 2010). The alignment bam files were then sorted and PCR duplicates were marked by MarkDuplicates. Variants were detected using the GATK pipeline following the best practices workflow (McKenna et al., 2010). The erroneous mismatches around small indels were realigned using IndelRealigner. The variants were called for each accession by GATK4 (4.2.0.0) HaploTypeCaller with parameter: emit-ref-confidence GVCF and individual GVCF files were merged using GenotypeGVCFs. SNPs were filtered based on the following criteria: (1) SNPs were filtered with “QD < 2.0| | FS > 60.0| | MQ < 40| | SOR > 3| | MQRankSum < −12.5| | ReadPosRankSum < −8.0”; (2) SNPs with read depth > 50 or < 2; (3) SNPs with missing rate > 10%; (4) variants with more than two alleles; (5) a minor allele frequency (MAF) of < 0.05; (6) SNPs within 5 bp of the closest Indel; and (7) SNPs in regions with a SNP count > 3 within 10 bp were all removed. The 187,728 SNPs located at fourfold-degenerate sites were further retrieved from the above-identified SNPs and then converted to phylip and aligned fasta format by a python script called vcf2phylip.py^2^. The maximum-likelihood trees were constructed using RAxML (RAxML-8.2.12) with the GTRGAMMA model (Stamatakis, 2014). The maximum-likelihood phylogenetic tree was visualized by using iTOL software (Letunic and Bork, 2021).

### Repeat Sequence Annotation

A total of 12 *Oryza* genomes were downloaded from the corresponding database for repeat sequence analysis (Supplementary Table 5). Repeat sequences in the *Oryza* genomes were identified by the following procedures. First, a *de novo* repeat library was constructed by using RepeatModeler (version 2.0.1) (Flynn et al., 2020). Furthermore, long terminal repeat (LTR) retrotransposons against the *Oryza* genome sequences were detected using LTRharvest, LTR_FINDER (version 1.07) (Xu and Wang, 2007), and LTR_retriever (version 2.8) (Ou and Jiang, 2018). The LTR retrotransposons found by the two methods were merged and the duplications were then removed, which were combined with other repeat sequences found by RepeatModeler (version 2.0.1) (Flynn et al., 2020) to form the preliminary repeat sequence library. The repeat sequences labeled “Unknown” in the repeat sequence library were further classified according to the best homology alignment (*E* value 1e−10) against the Rice TE Database^3^ (Copetti et al., 2015). Transposable elements (TEs) within each genome were identified by RepeatMasker (open-4-0-8) (Tarailo-Graovac and Chen, 2009) with the repeat sequence library established according to the above-mentioned steps. Pearson’s correlation was analyzed between lengths of repeat sequences and genome sizes. The graphs of correlation analysis were drawn using “ggplot2” and “ggpubr” packages in R (version 4.1.2).

### Classification and Insertion Time Estimation of Intact Long Terminal Repeats Retrotransposons

All intact LTR-RTs generated by LTR_retriever (version 2.8) (Ou and Jiang, 2018) were classified by TEsorter with default parameters (Zhang et al., 2022). The terminal repeat regions and other non-coding regions are the fastest evolving parts of TEs. According to the definition of family and subfamily (Wicker et al., 2007), we considered two intact LTR-RTs to belong to the same family if they share 80% (or higher) sequence identity within at least 80% of their long terminal repeat regions. The insertion times of intact LTR-RTs were estimated by following former studies (SanMiguel et al., 1998; Ma et al., 2004; Zhang and Gao, 2017). The two LTRs of each intact LTR-RTs were aligned using the MUSCLE multiple alignment method, and the Kimura 2-parameter method was used to calculate the distance (d) under the complete deletion option in MEGA11 (Tamura et al., 2021). The insertion time was then calculated by using *t* = d/2*r*, where the rate (*r*) of neutral evolution of 1.3 × 10^–8^ substitutions per site per year was used (Zhang and Gao, 2017).

## Results

### Optimization of DNA Flow Cytometry in the Genus *Oryza*

**FIGURE 1:**
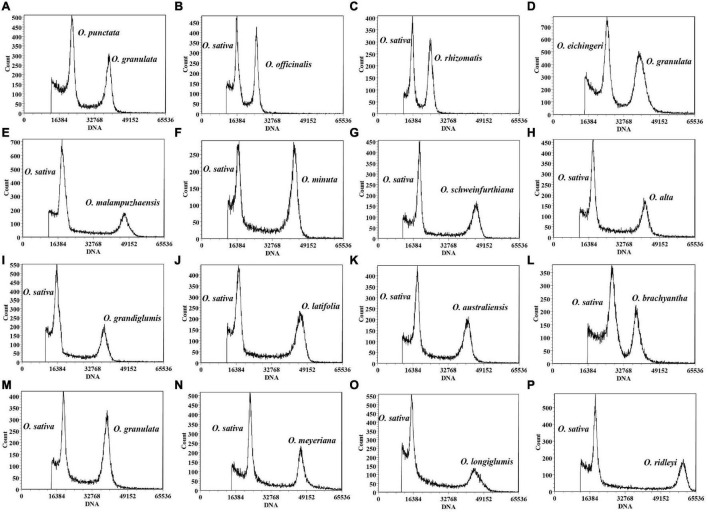
Flow cytometry histograms of *Oryza* species. *O. granulata* was used as an internal standard for *Oryza punctata* **(A)** and *Oryza eichingeri* **(D)**. *Oryza sativa* L. ssp. *japonica* cv. *Nipponbare* (0.7955 pg/2C) was employed as internal standard for *Oryza officinalis* **(B)**, *Oryza rhizomatis* **(C)**, *Oryza malampuzhaensis* **(E)**, *Oryza minuta* **(F)**, *Oryza schweinfurthiana* **(G)**, *Oryza alta* **(H)**, *Oryza grandiglumis* **(I)**, *Oryza latifolia* **(J)**, *Oryza australiensis* **(K)**, *Oryza brachyantha* **(L)**, *Oryza granulata* **(M)**, *Oryza meyeriana* **(N)**, *Oryza longiglumis* **(O)**, and *Oryza ridleyi* **(P)**.

An ideal DNA reference standard should have a genome size close to the target species, which could avoid the risk of non-linearity and offset errors (Bagwell et al., 1989). In this study, *O. sativa* L. ssp. *japonica* cv. *Nipponbare* with a DNA content of 0.7955 pg/2C (389 Mb/1C) (Sasaki, 2005), was employed as an internal standard. Nevertheless, the 2C peaks of *Oryza punctata* and *O. eichingeri* coincided with the *Nipponbare* peak, indicating that they might have similar DNA contents. Thus, *Nipponbare* was not an ideal internal standard to estimate genome sizes of *O. punctata* and *O. eichingeri.* Among the other non-AA species in *Oryza*, *O. granulata* was one of few species that were sequenced (Wu et al., 2018; Shi et al., 2020), whose genome size was approximately 1.5 times larger than those of *O. punctata* and *O. eichingeri*. It seems that *O. granulata* might serve as a suitable internal standard for estimating genome sizes of these two species by using flow cytometry analysis. In order to prevent the error caused by the two internal standards individually in the same experiment, genome sizes of *Nipponbare* and *O. granulata* were recalibrated against each other by detecting the intensity of fluorescence signals of the same number of cells in flow cytometry experiments. The nuclear DNA content of *O. granulata* (IRGC 80740) was 1.58 pg/2C, namely 772.62 Mb, when *O. sativa* L. ssp. *japonica* cv. *Nipponbare* was used as internal standard (Figure 1M and Supplementary Table 1), which was consistent with the sequenced *O. granulata* genome of approximately 777 Mb (Wu et al., 2018). Consequently, it is feasible to select *Nipponbare* or *O. granulata* as internal standards for genome sizes of different rice species.

Based on previous studies, we chose the bead beating method to prepare nuclear suspension with fresh rice leaves in Hanson’s nuclear isolation buffer (Hanson et al., 2005; Roberts, 2007; Cousin et al., 2009; Isidre et al., 2019). More than 10,000 stained nuclei were analyzed from the internal standard sample and target sample, respectively. It is much more than the previous studies that only analyzed 2,000 or 4,000 nuclei (Supplementary Table 3). Three repeated experiments for each sample were conducted to eliminate the systematic error. Eventually, credible results were acquired for 166 accessions from 16 non-AA genome species in the genus *Oryza* (Figure 1). The obtained estimates for half of the 16 species were close to one of the previously reported results except for *O. rhizomatis, O. eichingeri, Oryza grandiglumis, O. australiensis, Oryza meyeriana, O. granulata*, *O. brachyantha*, and *Oryza longiglumis*, which seemed to have been overestimated in earlier studies (Table 1). One of the major applications of genome size estimation is to evaluate the integrity of the assembled genomes. We compared the results of flow cytometry with the assembled genome lengths for the species that were sequenced in the genus *Oryza*. Our results showed that the estimated genome sizes in this study are fairly close to the assembled genome lengths, indicating high accuracy of the experimental assessment (Table 1).

**TABLE 1:** Summary of nuclear DNA contents of *Oryza* species estimated by using flow cytometry analysis.

Species	Genome type	Nuclear DNA content						
		This study				Previous data		
		Number of accessions	pg/2C ± SD	Range	Mb^a^/1C	pg/2C ± SD	Mb^b^/1C	References
*O. sativa*	AA					0.9; 0.88	434; 424	Cesar et al., 1994
						0.93; 0.91	449; 438	Uozu et al., 1997
							389^c^; 372^c^	Stein et al., 2018
							420^c^	Goff et al., 2002
							389^c^	Sasaki, 2005
							384^c^; 386^c^	Zhang et al., 2016
							390.3^c^	Du et al., 2017
							381.19^c^; 396^c^	Zhang et al., 2018
							377.6^c^	Jain et al., 2019
							367^c^	Tanaka et al., 2020
							386.5^c^; 383.6^c^	Choi et al., 2020
							391.56^c^; 395.77^c^	Song et al., 2021
							397.71^c^	Li et al., 2021
*O. rufipogon*	AA					0.95	459	Uozu et al., 1997
							450	Ammiraju et al., 2010
						0.91 ± 0.01	439	Ammiraju et al., 2006
						0.91 ± 0.06; 0.87 ± 0.08		Miyabayashi et al., 2007
							380.51^c^	Li et al., 2020a
							338^c^	Stein et al., 2018
*O. nivara*	AA						341.32; 375.01^c^	Zhang et al., 2014
						0.93 ± 0.01	448	Ammiraju et al., 2006
							338^c^	Stein et al., 2018
*O. barthii*	AA						370.17; 335.09^c^	Zhang et al., 2014
							411	Ammiraju et al., 2010
						0.94 ± 0.15; 0.95 ± 0.05		Miyabayashi et al., 2007
							308^c^	Stein et al., 2018
*O. glaberrima*	AA					0.73–0.76	352-366	Cesar et al., 1994
						0.87	420	Uozu et al., 1997
							316^c^	Wang et al., 2014
							380.44; 344.86^c^	Zhang et al., 2014
							354	Ammiraju et al., 2010
*O. glumaepatula*	AA					0.99	475	Uozu et al., 1997
							388.27; 334.67^c^	Zhang et al., 2014
						0.98 ± 0.03; 1.05 ± 0.06		Miyabayashi et al., 2007
							464	Ammiraju et al., 2010
							373^c^	Stein et al., 2018
*O. meridionalis*	AA					1.02	493	Uozu et al., 1997
							413.21; 340.78c	Zhang et al., 2014
						0.88 ± 0.07; 0.90 ± 0.04		Miyabayashi et al., 2007
							435	Ammiraju et al., 2010
							336^c^	Stein et al., 2018
*O. longistaminata*	AA					0.78	376	Cesar et al., 1994
							352	Ammiraju et al., 2010
						0.81	389	Uozu et al., 1997
						0.93 ± 0.08; 0.89 ± 0.07		Miyabayashi et al., 2007
						0.78	376	Cesar et al., 1994
							363.5^c^	Li et al., 2020b
							347^c^	Zhang et al., 2015
*O. punctata*	BB	27	0.90 ± 0.014	0.85–0.92	438	0.86 ± 0.17; 0.85 ± 0.08		Miyabayashi et al., 2007
						1.11	535	Uozu et al., 1997
						0.88 ± 0.18	425	Ammiraju et al., 2006
							394^c^	Stein et al., 2018
*O. officinalis*	CC	37	1.22 ± 0.029	1.14–1.26	597	1.35 ± 0.02	651	Ammiraju et al., 2006
						1.19 ± 0.05; 1.36 ± 0.20		Miyabayashi et al., 2007
						1.45	697	Uozu et al., 1997
							584^c^	Shenton et al., 2020
						1.14	550	Cesar et al., 1994
*O. eichingeri*	CC	15	1.01 ± 0.057	0.86–1.11	495	1.64 ± 0.08; 1.11 ± 0.05		Miyabayashi et al., 2007
						1.47	709	Uozu et al., 1997
							471^c^	Shenton et al., 2020
						1.17	564	Cesar et al., 1994
*O. rhizomatis*	CC	9	1.22 ± 0.009	1.20–1.24	597		559^c^	Shenton et al., 2020
						1.92 ± 0.17		Miyabayashi et al., 2007
*O. malampuzhaensis*	BBCC	2	1.99 ± 0.037	1.96–2.03	975			
*O. schweinfurthiana*	BBCC	20	1.90 ± 0.023	1.87–1.93	929	2.02 ± 0.04; 1.90 ± 0.13		Miyabayashi et al., 2007
*O. minuta*	BBCC	7	2.04 ± 0.020	2.00–2.07	998	1.67 ± 0.23; 1.92 ± 0.08		Miyabayashi et al., 2007
						2.33	1124	Cesar et al., 1994
*O. alta*	CCDD	6	1.88 ± 0.024	1.85–1.92	919	2.09 ± 0.019	1008	Ammiraju et al., 2006
						1.68 ± 0.33; 2.35 ± 0.14; 2.04 ± 0.06		Miyabayashi et al., 2007
							894.6^c^	Yu et al., 2021
O. grandiglumis	CCDD	7	1.84 ± 0.012	1.82–1.85	900	2.06 ± 0.05; 2.10 ± 0.18		Miyabayashi et al., 2007
						1.99	960	Cesar et al., 1994
*O. latifolia*	CCDD	8	2.15 ± 0.018	2.12–2.17	1051	2.32	1124	Cesar et al., 1994
						1.88 ± 0.01		Miyabayashi et al., 2007
*O. australiensis*	EE	8	1.85 ± 0.017	1.82–1.86	905	2.00 ± 0.8	965	Ammiraju et al., 2006
						1.99	960	Cesar et al., 1994
						1.96	946	Uozu et al., 1997
						1.92 ± 0.15; 1.93 ± 0.05		Miyabayashi et al., 2007
*O. brachyantha*	FF	2	0.57 ± 0.007	0.56–0.58	279	0.75 ± 0.07	362	Ammiraju et al., 2006
						0.72	346	Uozu et al., 1997
							261^c^	Chen et al., 2013
						0.63 ± 0.15; 0.60 ± 0.08		Miyabayashi et al., 2007
*O. granulata*	GG	7	1.59 ± 0.013	1.57–1.61	779	1.83 ± 0.28	882	Ammiraju et al., 2006
							672; 707; 736c	Shi et al., 2020
							777^c^	Wu et al., 2018
						2.29 ± 0.25; 2.46 ± 0.26		Miyabayashi et al., 2007
*O. meyeriana*	GG	4	1.60 ± 0.020	1.57–1.62	781	2.4 ± 0.24;2.27 ± 0.21		Miyabayashi et al., 2007
*O. ridleyi*	HHJJ	4	2.46 ± 0.047	2.40–2.53	1203	2.66 ± 0.14	1283	Ammiraju et al., 2006
						1.31; 1.85; 1.93	632-931	Cesar et al., 1994
						2.03 ± 0.33; 3.00 ± 0.23		Miyabayashi et al., 2007
*O. longiglumis*	HHJJ	3	2.34 ± 0.041	2.29–2.39	1144	2.72 ± 0.21; 2.91 ± 0.15		Miyabayashi et al., 2007
*O. coarctata*	KKLL						665	Mondal et al., 2017
							569.9^c^	Mondal et al., 2018
							771	Ammiraju et al., 2010
							573^c^	Bansal et al., 2021

*^a^1 pg = 978 Mb (Dolezel et al., 2003), which was used to convert pg to Mb in this study. ^b^1 pg = 965 Mb, which was used to convert pg to Mb in previous studies. ^c^These values represent lengths of the assembled genomes.*

### Comparisons of Genome Sizes Estimated by Flow Cytometry and *k*-mer Analyses

The flow cytometry analysis largely relies on a species with a well-documented genome size as an internal standard to accurately estimate genome sizes. With the development of genome sequencing technologies, a computational method to estimate genome size, which is independent of internal standards, has been extensively applied to more and more plant species based on the *k*-mer frequency of whole-genome sequencing data (Chen et al., 2015; Guo et al., 2015; He et al., 2016). In order to comprehensively investigate genome size variation in *Oryza* species and further verify the accuracy of the obtained flow cytometry results, we resequenced two accessions for each examined species to calculate genome sizes by using *k*-mer analysis. Our results showed that genome sizes estimated by gce (gce-v1.0.2, gce-alternative) were slightly larger than those through flow cytometry analyses for most species except for *Oryza malampuzhaensis* (IRGC 100957), *Oryza minuta* (IRGC 105131), *O. longiglumis* (IRGC 105147), *O. longiglumis* (IRGC 100974), *O. ridleyi* (IRGC 100821), and *O. granulata* (IRGC 102117) in this study (Table 2). Note that the genome size of *O. granulata* (IRGC 102117) estimated by using flow cytometry analysis was adopted from Wu et al. (2018) since fresh leaves were not available in this study. We employed the flow cytometry analysis to estimate genome sizes of seven accessions of *O. granulata*, and the average genome size of *O. granulata* was approximately 779 Mb, which was close to the result calculated by *k*-mer analysis (Table 1 and Supplementary Table 1). Comparisons of genome sizes estimated through flow cytometry and *k*-mer analyses showed that the obtained results varied about 10% and less than 5% in more than half of those species.

**TABLE 2:** Genome sizes of *Oryza* species estimated by using flow cytometry and *k*-mer analyses.

Species	Genome type	IRGC No.	Genome size estimated by flow cytometry analysis (Mb)	Genome size estimated by *k*-mer analysis (Mb)
*O. punctata*	BB	99575	437.0	462.1
		104974	440.5	466.2
*O. officinalis*	CC	105099	590.7	613.2
		80760	581.4	592.7
*O. eichingeri*	CC	89245	504.4	517.7
		89246	499.4	535.2
*O. rhizomatis*	CC	103414	595.2	611.0
		103410	596.5	616.9
*O. schweinfurthiana*	BBCC	105137	959.0	1003.6
		100886	922.2	923.7
*O. malampuzhaensis*	BBCC	80767	956.5	990.9
		100957	993.0	938.1
*O. minuta*	BBCC	105131	1004.7	1022.4
		105126	988.9	1043.1
*O. alta*	CCDD	100161	910.5	955.2
		105222	904.5	940.5
*O. grandiglumis*	CCDD	105669	905.6	934.4
		106241	902.1	963.9
*O. latifolia*	CCDD	102481	1053.3	1096.7
		101392	1060.7	1144.0
*O. australiensis*	EE	105278	904.5	929.4
		105274	904.8	931.0
*O. brachyantha*	FF	105151	278.5	287.7
		101236	284.9	287.6
*O. longiglumis*	HHJJ	105147	1169.2	1040.2
		100974	1100.0	956.2
*O. ridleyi*	HHJJ	100821	1214.2	1206.2
		100877	1191.1	1258.5
*O. granulata*	GG	102117	882^a^	719.2
		NA	782.7	732.3
*O. meyeriana*	GG	104989	794.3	800.6
		106474	766.3	814.2
*O. coarctata*	KKLL	NA	665^b^	554.9

*^a^The genome size of O. granulata (IRGC 102117) estimated by using flow cytometry was adopted from Wu et al. (2018). ^b^The genome size of O. coarctata estimated by flow cytometry was adopted from Mondal et al. (2017, 2018). NA represents not available.*

### Genome Size Variation Across *Oryza* Genome Types and Species

Flow cytometry analyses were used to estimate DNA contents of 166 accessions from 16 *Oryza* species throughout the world (Supplementary Table 1). Our results indicated that the 2C DNA contents in the genus *Oryza* varied nearly 4.3-fold, ranging from approximately 0.57 pg in *O. brachyantha* to approximately 2.46 pg in *O. ridleyi* (Table 1). The examined accessions belonging to the same species seemingly had similar DNA contents. Notably, *O. eichingeri* harbored the largest intraspecific genome size variation (Table 1), whose 2C DNA contents ranged from approximately 0.86–1.11 pg/2C among 15 accessions, and the standard deviation (SD) value of 15 accessions was approximately 0.057, while the SD value of other species was lower than 0.05 (Table 1). Our results are consistent with the previous observation that species with the same genome type usually had similar genome sizes in the genus *Oryza* (Miyabayashi et al., 2007), but slight differences still existed within the species with CC, BBCC, and CCDD genome types. The 2C DNA content of *O. eichingeri* (CC) was 0.11 pg (about 100 Mb) lower than the other two CC genome type species. Similarly, the 2C DNA contents of *Oryza schweinfuriana* (BBCC) were somewhat lower than the other two BBCC genome type species. The genome size of *O. malampuzhaensis* (BBCC) (approximately 975 Mb) was first reported in this study. *Oryza latifolia* was approximately 100 Mb larger than the other two CCDD genome type species in genome size (Table 1).

**FIGURE 2:**
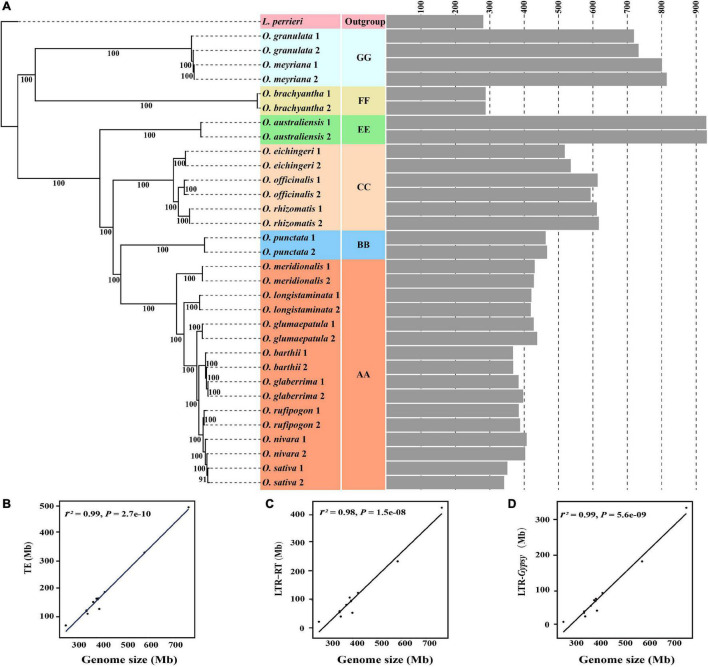
Evolutionary dynamics of genome sizes and transposable elements across the diploid *Oryza* species. The phylogenetic tree was constructed based on the SNPs located on fourfold-degenerate sites **(A)**. The gray bar graph represents the genome size estimated by using *k*-mer analysis. The number of branches represents the bootstrap values. The correlation is shown between genome sizes and TEs **(B)**, LTR-RTs **(C)**, and LTR/*Gypsy*-RTs **(D)**, respectively.

Combined with previous studies on the genome size of AA genome type species (Sasaki, 2005; Zhang et al., 2014, 2015; Li et al., 2020a; Xie et al., 2021), our results showed that genome sizes among the six diploid genome types in *Oryza* varied nearly threefold, ranging from approximately 279 Mb (FF) to approximately 905 Mb (EE) (Table 1). *O. brachyantha* (FF) harbors the smallest genome in the genus *Oryza*, while *O. australiensis* (EE) has the largest genome size among diploid species, which is even close to DNA contents of the tetraploid species with BBCC and CCDD genome types (Figure 2 and Table 1). Among the six diploid genome types, the species with the BB genome type had relatively similar genome sizes to the AA genome type species (Figure 2). The species with GG- genome had genome size nearly two times larger than AA genome species (Figure 2).

Genome sizes of the tetraploid species in the genus *Oryza* varied nearly twofold, ranging from approximately 570 Mb (*O*ryza *coarctata*) to approximately 1,203 Mb (*O. ridleyi*) (Table 1). The previously reported flow cytometry data showed that the genome size of *O*. *coarctata* was approximately 665 Mb (Mondal et al., 2017) or approximately 771 Mb (Ammiraju et al., 2010), while the assembled genome length was approximately 570 Mb (Mondal et al., 2018; Bansal et al., 2021). We further used *k*-mer analysis to estimate its genome size to be approximately 555 Mb, which was the same as the diploid species with CC genome type (Table 2). Therefore, *O*. *coarctata* was the smallest in genome size among the tetraploid species. The tetraploid species with BBCC- and CCDD genome types had similar genome sizes of approximately 900–1,000 Mb (Table 1). Interestingly, the genome size of species with BBCC genome types was approximately equal to the totaling genome sizes of species with BB and CC genome types. The genome size of *O. ridleyi* was about 1,203 Mb, which was the largest among the *Oryza* species with known genome sizes. It was two times larger than *O*. *coarctata* and approximately 60 Mb larger than another HHJJ genome species, *O. longiglumis* (Table 1).

Our results altogether showed that the genome size largely varied nearly 4.3-fold in the genus *Oryza*, threefold among the diploid species, and twofold among the tetraploid species. Genome sizes slightly vary within the species, and species with the same genome type usually have similar DNA contents. Nevertheless, the interspecific genome size variation among species with the same genome type is still larger than the intraspecific genome size variation. It is worth noting that the genome sizes of the tetraploid species are not always two times larger than those of the diploid species, and genome sizes of some diploids are even larger than those of tetraploids. For example, both *O. granulata* and *O. australiensis* had a larger genome size than the tetraploid *O*. *coarctata* (Table 1).

The obtained results of genome sizes in this study provide us an opportunity to reclassify unidentified *Oryza* accessions. The tetraploid populations of *O. officinalis* (formerly identified as *O. officinalis*) and the tetraploid populations of *O. punctata* (formerly identified as *O. punctata*) were classified as *O. malampuzhaensis* and *O. schweinfurthiana*, respectively (Sano, 1980; Vaughan, 1989; Li et al., 2001). For example, IRGC 100957 and IRGC 80767 were classified as *O. malampuzhaensis*, which were formerly regarded as *O. officinalis* (Supplementary Table 1). Meanwhile, 20 accessions, such as IRGC 101439 and IRGC 88827, were formerly identified as *O. punctata*, but they were all updated to be *O. schweinfurthiana* in International Rice Genebank^4^ (Supplementary Table 1). In this study, the flow cytometry analysis of these accessions confirmed that they were tetraploid (Supplementary Table 1). Moreover, 2C DNA contents of the other 11 *O. punctata* accessions (IRGC 100881, IRGC 88825, IRGC 100177, IRGC 100892, IRGC 101429, IRGC 105082, IRGC 105128, IRGC 105160, IRGC 105174, IRGC 105181, and IRGC 105182) were approximately 1.9 pg, which are two times larger than that of the diploid *O. punctata*. Thus, these 11 accessions were likely to be the tetraploid *O. schweinfurthiana* (Table 3). IRGC 105321 was earlier identified as *O. officinalis* in International Rice Genebank, while 2C DNA content was measured to be approximately 2.02 pg, indicating that IRGC 105321 might be classified as *O. malampuzhaensis* (Table 3).

**TABLE 3:** Reclassification of *Oryza* accessions based on flow cytometry analysis.

IRGC No.	Former classification^a^		Source country	DNA content		Current classification^b^	
	Species	Genome type		2C/pg ± SD	1C/Mbp	Species	Genome type
100881	*O. punctata*	BB	NA	1.87 ± 0.011	913.87	*O. schweinfurthiana*	BBCC
88825	*O. punctata*	BB	Madagascar	1.89 ± 0.006	921.91	*O. schweinfurthiana*	BBCC
100177	*O. punctata*	BB	NA	1.87 ± 0.008	916.40	*O. schweinfurthiana*	BBCC
100892	*O. punctata*	BB	NA	1.90 ± 0.006	929.99	*O. schweinfurthiana*	BBCC
101429	*O. punctata*	BB	Uganda	1.90 ± 0.010	927.07	*O. schweinfurthiana*	BBCC
105082	*O. punctata*	BB	Philippines	1.89 ± 0.008	926.12	*O. schweinfurthiana*	BBCC
105128	*O. punctata*	BB	Philippines	1.89 ± 0.010	926.38	*O. schweinfurthiana*	BBCC
105160	*O. punctata*	BB	Uganda	1.88 ± 0.011	919.55	*O. schweinfurthiana*	BBCC
105174	*O. punctata*	BB	Malaysia	1.90 ± 0.014	928.32	*O. schweinfurthiana*	BBCC
105181	*O. punctata*	BB	Uganda	1.85 ± 0.008	905.07	*O. schweinfurthiana*	BBCC
105182	*O. punctata*	BB	Uganda	1.85 ± 0.005	902.83	*O. schweinfurthiana*	BBCC
105321	*O. officinalis*	CC	India	2.02 ± 0.017	988.78	*O. malampuzhaensis*	BBCC

*^a^Classified by International Rice Genebank. ^b^Reclassification based on genome sizes estimated by flow cytometry analysis. NA represents not available.*

### Evolutionary Dynamics of Genome Sizes and Transposable Elements Across the *Oryza* Species

To examine the evolutionary dynamics of genome sizes in the genus *Oryza*, a credible phylogenetic tree with almost 100% bootstrap support was reconstructed based on SNPs located on fourfold-degenerate sites (Figure 2A). All of the *Oryza* diploid species were divided into the two main clades, of which one comprised the species with AA, BB, CC, and EE genome types, while the other included species with FF and GG genome types (Figure 2A). The result is congruent with the topology of the phylogenetic tree based on chloroplast genomes (Gao et al., 2019). Except that the genome sizes varied nearly 2.5-fold between FF and GG genome type species, we failed to find sufficient evidence to support the correlation between the genome sizes and phylogenetic relationships in the clade with FF and GG genome types due to including too few species. However, it is noteworthy that genome size gradually decreased during the evolution of the clades with AA, BB, CC, and EE genome types. The genome size quickly decreased from EE genome type (approximately 900 Mb) to CC genome type (approximately 600 Mb), and then the decreasing trend gradually slowed down from CC to BB genome types and BB to AA genome types. Even among the eight species with AA genome type, the genome size tended to decrease slowly with the phylogenetic relationships. Consequently, it is reasonable to presume that there are some kind of correlation between genome sizes and phylogenetic relationships in the genus *Oryza*.

Considering that transposable elements play an important role in rice genome expansion (Dodsworth et al., 2015; Suh, 2019), we annotated and compared the contents of all types of TEs from 12 high-quality *Oryza* genomes to investigate the causes of genome size variation in the genus *Oryza* (Supplementary Table 5). Our results showed that, among all types of TEs, LTR-RTs and especially LTR/*Gypsy* RTs greatly varied across the twelve investigated *Oryza* genomes (Supplementary Table 4). Our further analysis apparently revealed that TEs, LTR-RTs, and LTR/*Gypsy* RTs were all significantly correlated with genome sizes (Figures 2B–D).

**FIGURE 3:**
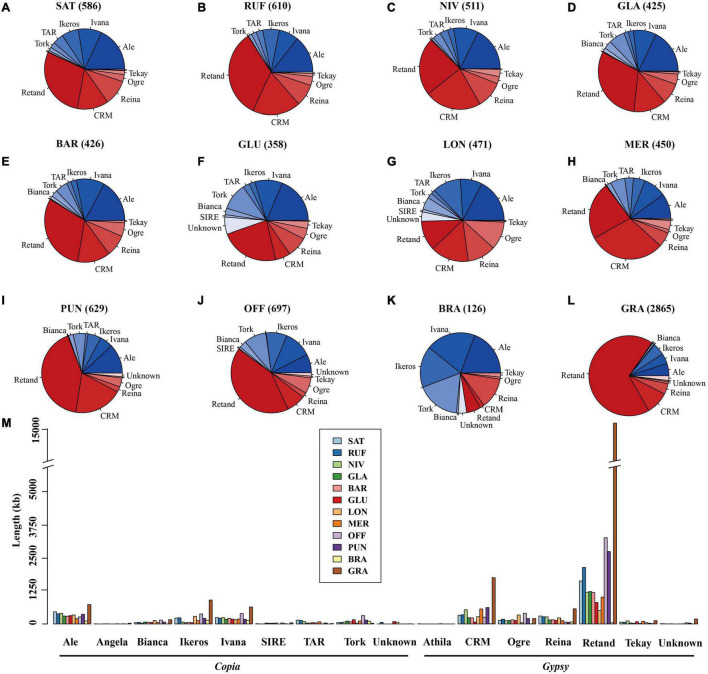
Copy number **(A–L)** and length **(M)** of intact LTR-RTs in different lineages of the *Oryza* genomes. **(A–L)** Shows the classification and proportions of intact LTR-RTs in corresponding species; the lineages belonging to the *Gypsy* and *Copia* superfamilies are shown in red and blue color, respectively. The numbers in brackets represent the copy number of intact LTR-RTs. **(M)** Shows the length of intact LTR-RTs of different lineages in the *Oryza* genomes. SAT, NIV, RUF, GLA, BAR, GLU, LON, MER, PUN, OFF, BRA, and GRA represent *O. sativa*, *O. nivara*, *O. rufipogon*, *O. barthii*, *O. glaberrima*, *O. glumaepatula*, *O. longistaminata*, *O. meridionalis*, *O. punctata*, *O. officinalis*, *O. brachyantha*, and *O. granulata*, respectively.

**FIGURE 4:**
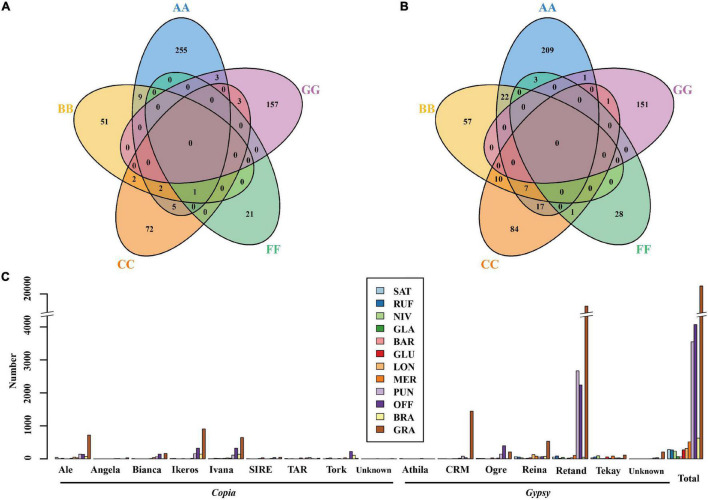
Venn diagrams of LTR-RT families belonging to the *Gypsy* **(A)** and *Copia* **(B)** superfamilies among the five (AA-, BB- CC-, FF-, GG-) rice genome types, and lengths of species-specific intact LTR-RTs of the 12 *Oryza* genomes **(C)**.

**FIGURE 5:**
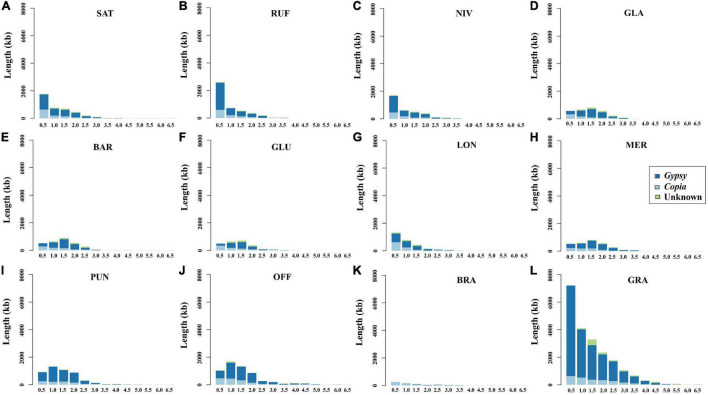
Length of intact LTR-RTs inserted in the twelve examined *Oryza* genomes at different time periods. The values on the abscissa represent the insertion time span of the intact LTR-RTs, and the adjacent values take 0.5 as an interval value. For example, 1 represents the time period from 0.5 to 1 million years ago (MYA). **(A–L)** Represents *O. sativa*, *O. nivara*, *O. rufipogon*, *O. barthii*, *O. glaberrima*, *O. glumaepatula*, *O. longistaminata*, *O. meridionalis*, *O. punctata*, *O. officinalis*, *O. brachyantha*, and *O. granulata*, respectively.

In order to examine the evolutionary dynamics of LTR-RTs that determine rice genome size variation, 8,154 intact LTR-RTs from the twelve *Oryza* genomes, belonging to AA-, BB-, CC-, FF-, and GG genome types, were detected and classified by TEsorter (Zhang et al., 2022; Figure 3). Of them, *Ale* is the lineage with the most abundant and the longest length of intact LTR RTs of the *Copia* superfamily among AA, BB, and GG genome types, while the *Ivana* lineage harbored the most intact LTR-RTs in *O. officinalis* and *O. brachyantha* (Figure 3). Our results showed that the total length of intact LTR-RTs belonging to the *Retand* lineage was the longest lineage in *Oryza* species except for *O. brachyantha*, which possessed the smallest genome size in *Oryza*. In sharp contrast to the other eleven *Oryza* species, *O. brachyantha* interestingly possessed much more number and longer length of intact LTR-RTs belonging to the *Copia* superfamily than those belonging to the *Gypsy* superfamily (Figure 3). According to similarities of LTR sequences, 8,154 intact LTR-RTs obtained from twelve *Oryza* species were classified into 1,305 families, of which 591 families belonged to the *Copia* superfamily, 581 families belonged to the *Gypsy* superfamily, and the remaining 133 families could not be classified into neither of the two superfamilies. Although there were fewer *Gypsy* families than the *Copia* families, the number of intact LTR-RTs of *Gypsy* families was two times more than that of the *Copia* families (Supplementary Table 6). Our results showed that there were no shared LTR-RT families among the examined genome types, suggesting rapid turnover of LTR-RTs across the *Oryza* genomes (Figures 4A,B). Total lengths of species-specific intact LTR-RTs in *O. granulata*, *O. officinalis*, and *O. punctata* were much larger than that of other species, especially in *O. granulata*, which possessed the largest genome size among the 12 analyzed *Oryza* species, indicating that species-specific LTR-RTs has made a great contribution to its large genome size (Figure 4C). Further analysis of lineage-specific intact LTR-RTs showed that the amplification of the *Retand* lineage may account for larger genome sizes of *O. granulata*, *O. officinalis*, and *O. punctata* (Figure 4C). By comparing lengths of intact LTR-RTs inserted within 6.5 million years (MYR), we found that the inserted intact LTR-RTs belonging to the *Gypsy* superfamily were longer than the *Copia* superfamily in *Oryza* species except for *O. brachyantha*. However, the inserted intact LTR-RTs belonging to the *Copia* supfamily were longer than the *Gypsy* superfamily in species *O. glaberrima*, *O. barthii*, *O. glumeaputala*, and *O. longistaminata* within 0.5 million years ago (Figure 5). Our results showed that the examined species may have experienced dissimilar amplification histories of retrotransposons, resulting in remarkably different genome sizes (Figure 5). Our findings also indicate that the closely related rice species, such as *O. sativa* and its wild ancestors (*O. nivara* and *O. rufipogon*), and *O. glaberrima* and its wild ancestor *O. barthii*, may have experienced similar amplification history of retrotransposons (Figures 2, 3, 5).

## Discussion

### Optimizing Flow Cytometry Conditions to Estimate Genome Sizes

The genus *Oryza* has attracted great attention for its huge economic and humanistic values. With the rapid development of sequencing technologies, recent decades have witnessed great progress in comparative genomics of the genus and especially AA genome type species (Zhang et al., 2014; Stein et al., 2018; Zhao et al., 2018; Li et al., 2020a,c; Shi et al., 2020). As an important step to survey the genome size before whole-genome sequencing, to date, continuous efforts have been put to estimate genome sizes of the *Oryza* species by using flow cytometry analysis (Cesar et al., 1994; Uozu et al., 1997; Ammiraju et al., 2006; Miyabayashi et al., 2007), resulting in inconsistent results. For example, the DNA content of *O. latifolia* was estimated to be 2.32 pg/2C (Cesar et al., 1994), while it was assessed to be 1.88 pg/2C (Miyabayashi et al., 2007). The DNA contents of other species, *O. granulata*, were measured to be 2.29 pg/2C or 2.49 pg/2C (Miyabayashi et al., 2007), while it was reported to be 1.83 pg/2C (Ammiraju et al., 2006) and slight discrepancy was reported in many other species.

We compared the results obtained in this study with those previously reported data to figure out the possible reasons for the inconsistency in the genome size assessment. The first factor generating different results may come from internal standards and cognition of internal standard genome sizes. Earlier studies usually employed chicken red blood (CRBC) as an internal standard, while the genome sizes of CRBC used for the calculation were different in different studies (Cesar et al., 1994; Uozu et al., 1997; Miyabayashi et al., 2007). Subsequently, the sequenced *Nipponbare* was often used as an internal or external standard (0.91 pg/2C) for flow cytometry experiments in the genus *Oryza* (Ammiraju et al., 2006; Miyabayashi et al., 2007), while the generation of high-quality *Nipponbare* reference genome sequence makes us widely recognize its genome size to be 389 Mb (Sasaki, 2005). If the formula, 1 pg DNA = 978 Mb, was used to convert base pairs (bp) to picograms (pg), the DNA content of *Nipponbare* should be approximately 0.7955 pg/2C, instead of 0.91 pg/2C. It is worth noting that the formula, 1 pg DNA = 965 Mb, was used in almost all previous studies (Cesar et al., 1994; Uozu et al., 1997; Ammiraju et al., 2006; Miyabayashi et al., 2007), while Dolezel et al. (2003) suggested that the formula, 1 pg DNA = 978 Mb, maybe more accurate, which was verified in many species (Huang et al., 2013; Tyagi et al., 2019). The other major factor that may affect the estimated genome size by flow cytometry analysis was the standardization, including external and internal standardization. The nuclei of the sample and standard were separately isolated, stained, and analyzed if the reference standard acts as an external standard, and the generated results may be suspicious, resulting from the discrepancy in the process of sample separation and staining. Even if the parameter settings of the instrument remain unchanged, the position of the 2C peak of the sample may also shift due to random drift of the instrument. However, these shortcomings may be avoided by internal standardization, in which the nuclei of the sample and standard were simultaneously isolated, stained, and analyzed (Dolezel and Bartos, 2005). Strictly speaking, the use of external standards thus leads to more or fewer errors, while the application of internal standards can generate relatively accurate results. In addition, enough cells should be detected in flow cytometry experiments to ensure the accuracy of the results, and 5,000–20,000 nuclei were needed for each sample (Galbraith et al., 1997). However, only 2,000–4,000 nuclei were analyzed in *Oryza* by using flow cytometry analysis in previous studies (Cesar et al., 1994; Uozu et al., 1997; Ammiraju et al., 2006; Miyabayashi et al., 2007). Therefore, it is necessary to employ unified standards to further update genome size estimates of the *Oryza* species according to the latest documentation of internal reference genome *Nipponbare*.

To obtain accurate data, the affected factors described above must be corrected in the flow cytometry experiments. In this study, we employed *Nipponbare* (0.7955 pg/2C, 389 Mb/1C) and *O. granulata* (1.5812 pg/2C, 773 Mb/1C) as internal standards upon genome size variation of the target species. These two standards were calibrated against each other to avoid errors caused using different internal standards. The updated DNA content of *Nipponbare* (0.7955 pg/2C, 389 Mb/1C) and the correct conversion formula were used to calculate the DNA contents of the target species, and up to approximately 10,000 nuclei were collected from each sample to ensure high-quality data. Simultaneously, we compared to flow cytometry experimental procedures and statistical methods, showing that the obtained genome sizes via these two methods varied from about 10% to less than 5% in more than half of the examined species. Our results also showed that genome sizes of *O. granulata, O. brachyantha, O. rhizomatis, O. eichingeri, O. officinalis, O. punctata*, and *O. alta* are fairly close to the assembled genome lengths (Chen et al., 2013; Stein et al., 2018; Wu et al., 2018; Shenton et al., 2020; Yu et al., 2021), strongly supporting the consistency and accuracy of our flow cytometry experiments. Thus, genome sizes estimated by using flow cytometry analysis can be reliably used for the species identification with the obtained genome sizes. Based on the measurement of genome sizes, *O. malampuzhaensis* and *O. schweinfurthiana* can be clearly identified from the species formerly classified as *O. officinalis* and *O. punctata*, respectively.

### Genome Size Variation and Evolution of *Oryza* Allotetraploids and Their Diploid Progenitors

Allopolyploids were usually thought to be caused by hybridization combined with genome doubling (Doyle et al., 2008; Soltis et al., 2014). In the genus *Oryza*, there are five tetraploid genome types (BBCC, CCDD, HHJJ, HHKK, and KKLL), which originated from hybridization events. It was suggested that the tetraploid BBCC- species originated independently (Bao et al., 2006). Zou et al. (2015) proposed that the diploid *O. punctata* (BB) and *O. officinalis* (CC) were the parental progenitors of *O. minuta* and *O. malampuzhaensis*, whereas the diploid *O. punctata* (BB) and *O. eichingeri* (CC) were the progenitors of *O. schweinfurthiana.* Our results showed that genome sizes of the three tetraploids with BBCC genome type were almost equal to totaling genome sizes of their diploid donor species. For instance, the 2C DNA content of *O. minuta* (BBCC, 2.04 pg/2C) and *O. malampuzhaensis* (BBCC, 1.99 pg/2C) was approximately equal to the sum of the 2C DNA contents of their parental progenitors, *O. punctata* (BB, 0.90 pg/2C) and *O. officinalis* (CC, 1.22 pg/2C). Similarly, the 2C DNA content of *O. schweinfurthiana* (BBCC, 1.9 pg/2C) was equal to the sum of *O. punctata* (BB, 0.90 pg/2C) and *O. eichingeri* (CC, 1.01 pg/2C). Among the three tetraploid species with CCDD genome type, *O. alta* (CCDD, 1.88 pg/2C) and *O. grandiglumis* (CCDD, 1.84 pg/2C) basically had the similar 2C DNA contents, while *O. latifolia* (CCDD, 2.15 pg/2C) was larger than both of them in genome size. Since the diploid species with DD genome species, which might be extinct, have not been found in nature, CCDD genome species were proposed to originate from a single allopolyploidization event with CC genome as their maternal parent while EE genome species might serve as the paternal donor (Ge et al., 1999; Bao and Ge, 2004). However, our results showed that the genome size of *O. australiensis* with EE genome (1.85 pg/2C) was similar to that of CCDD genome species (*O. alta*, 1.88 pg/2C; *O. grandiglumis*, 1.84 pg/2C; *O. latifolia*, 2.15 pg/2C). Although multiple pieces of evidence supported that EE genome and CCDD genome were closely related, it is questionable that EE genome served as the diploid donor of CCDD genome unless an extremely large-scale DNA loss occurred after speciation. As for the tetraploid KKLL, HHJJ, and HHKK genome species, the diploid donor species with HH, KK, and LL genomes have not been identified yet. The *Oryza* phylogeny constructed by using *Adh1*, *Adh2*, and *mat*K gene fragments failed to detect evident phylogenetic relationships among these genomes in the context of existing diploid species (Ge et al., 1999). Hence, the mechanism determining genome size variation in these tetraploid species is still an opening question to address, which is waiting for their genomes to be sequenced. Considering the formation mechanism of BBCC genome size, polyploidy results in approximately doubling the genome size, and the genome size of tetraploid species is supposed to be the sum of the parent genome size unless large-scale deletion events occurred after speciation.

### Long Terminal Repeat Retrotransposons Serve as Drivers of Rice Genome Size Evolution

It has been demonstrated that rapid amplification of TEs and particularly LTR retrotransposons play an important role in rice genome expansion (McCarthy et al., 2002; Gao et al., 2004; Vitte et al., 2007; Zuccolo et al., 2007; Zhang and Gao, 2017). In this study, flow cytometry and *k*-mer analyses indicated that 2-fold genome size variation existed among the tetraploid *Oryza* species, while the diploid species varied more than threefold, ranging from approximately 279 Mb in *O. brachyantha* (FF) to approximately 905 Mb in *O. australiensis* (EE). We also observed a significant correlation between genome sizes and phylogenetic relationships among diploid species, and genome sizes exhibited a gradually decreased trend during the evolutionary process in the clade with AA, BB, CC, and EE genome types. Our comparative genomic analyses revealed that proportions of LTR retrotransposons and especially LTR/*Gypsy* retrotransposons varied greatly across diploid rice genomes with AA, BB, CC, FF, and GG genome types, ranging from 5.24% in *O. brachyantha* (FF-) to 44.54% in *O. granulata* (GG). Moreover, our results clearly showed that TEs, LTR-RTs, and LTR/*Gypsy* RTs were all significantly correlated with genome sizes. Among all six diploid genome types of *Oryza*, only EE genome type species *O. australiensis* has not been sequenced. It was reported that, however, three LTR retrotransposon families accounted for more than 60% of the *O. australiensis* genome, two of which belong to the *gypsy* superfamily, accounting for about 35% of the genome (Piegu et al., 2006). Thus, the amplification of LTR retrotransposons and particularly LTR/*gypsy* retrotransposons may largely account for genome size variation, becoming the major driving force in the genus *Oryza*. We failed to find any shared LTR retrotransposon families among *Oryza* species with different genome types, and early divergent species, such as GG, CC, and BB genome species, owned more species-specific families than those AA genome species, indicating rapid evolution of LTR retrotransposons in *Oryza* (Vitte et al., 2007; Zhang and Gao, 2017). LTR retrotransposons have been proven to undergo bursts of amplification within the past 5 Myr, and the half-life of LTR retrotransposon sequences in the rice genome was estimated to be less than 3 Myr (Vitte et al., 2007). As claimed in previous studies, the unequal homologous recombination and illegitimate recombination were primarily responsible for the removal of LTR-retrotransposons, and unequal homologous recombination had been more efficient at purging extraneous DNA (Ma et al., 2004; Tian et al., 2009). Most plant genomes in nature have undergone the polyploidization process and then rapidly complete the diploid process through large-scale chromatin rearrangement and deletion events so as to stabilize the genome expansion (Blanc and Wolfe, 2004); such events also occurred in rice (Wang et al., 2005). It is our belief that the genome size evolution in the genus *Oryza* has been a long and ongoing process to adapt to global environmental changes, making *Oryza* become an excellent model to address how polyploidization and TE dynamics together drive the genome size variation and evolution in plants.

## Conclusion

We accurately estimated genome sizes of 166 accessions belonging to 16 non-AA genome *Oryza* species using flow cytometry and *k*-mer analyses. Our results showed that genome sizes largely varied approximately fourfold in the genus *Oryza*, ranging from approximately 279 Mb in *O. brachyantha* (FF) to approximately 1,203 Mb in *O. ridleyi* (HHJJ), revealing a gradually decreased trend during the evolutionary process in the clade with AA, BB, CC, and EE genome types. We found that the contents of TEs, LTR retrotransposons, and LTR/*Gypsy* retrotransposons varied greatly but they significantly correlated with genome sizes. Although the species with different rice genome types may have experienced dissimilar amplification histories of retrotransposons, resulting in remarkably different genome sizes, the closely related rice species may have experienced similar amplification history. Thus, the amplification of LTR retrotransposons and particularly LTR/*gypsy* retrotransposons largely account for genome size variation in the genus *Oryza*.

## Data Availability Statement

The data presented in this study are deposited in the NCBI Sequence Read Archive (SRA) repository, accession number PRJNA833653.

## Author Contributions

L-ZG designed the study. S-FD, X-GZ, G-RH, J-YL, J-QT, and X-HJ executed the experiment. S-FD and DZ performed the data analyses. S-FD drafted the first manuscript. L-ZG and S-FD revised the final manuscript. All authors contributed to the article and approved the submitted version.

## Conflict of Interest

The authors declare that the research was conducted in the absence of any commercial or financial relationships that could be construed as a potential conflict of interest.

## Publisher’s Note

All claims expressed in this article are solely those of the authors and do not necessarily represent those of their affiliated organizations, or those of the publisher, the editors and the reviewers. Any product that may be evaluated in this article, or claim that may be made by its manufacturer, is not guaranteed or endorsed by the publisher.

## Footnotes

1
https://cvalues.science.kew.org/

2
https://github.com/edgardomortiz/vcf2phylip

3
http://omap.org/cgi-bin/rite/index.cgi

4
https://gringlobal.irri.org/gringlobal/search

## References

[B1] Ammiraju J. S. S. Song X. Luo M. Sisneros N. Angelova A. Kudrna D. (2010). The *Oryza* BAC resource: a genus-wide and genome scale tool for exploring rice genome evolution and leveraging useful genetic diversity from wild relatives. *Breeding Sci.* 60 536–543. 10.1270/jsbbs.60.536 26081539

[B2] Ammiraju J. S. Luo M. Goicoechea J. L. Wang W. Kudrna D. Mueller C. (2006). The *Oryza* bacterial artificial chromosome library resource: construction and analysis of 12 deep-coverage large-insert BAC libraries that represent the 10 genome types of the genus *Oryza*. *Genome Res.* 16 140–147. 10.1101/gr.3766306 16344555 PMC1356138

[B3] Bagwell C. B. Baker D. Whetstone S. Munson M. Hitchcox S. Ault K. A. (1989). A simple and rapid method for determining the linearity of a flow cytometer amplification system. *Cytom. Part A* 10 689–694. 10.1002/cyto.990100604 2582958

[B4] Bansal J. Gupta K. Rajkumar M. S. Garg R. Jain M. (2021). Draft genome and transcriptome analyses of halophyte rice *Oryza coarctata* provide resources for salinity and submergence stress response factors. *Physiol. Plantarum* 173 1309–1322. 10.1111/ppl.13284 33215706

[B5] Bao Y. Ge S. (2004). Origin and phylogeny of *Oryza species* with the CD genome based on multiple-gene sequence data. *Plant Syst. Evol.* 249 55–66. 10.1007/s00606-004-0173-8

[B6] Bao Y. Zhou H. F. De Yuan Hong Ge S. (2006). Genetic diversity and evolutionary relationships of *Oryza species* with the B- and C-genomes as revealed by SSR markers. *J. Integr. Plant Biol.* 49 339–347. 10.1007/BF03178809

[B7] Bennett M. D. Leitch I. J. (2005). Nuclear DNA amounts in angiosperms: progress, problems and prospects. *Ann. Bot.* 95 45–90. 10.1093/aob/mci003 15596457 PMC4246708

[B8] Bennett M. D. Price H. J. Johnston J. S. (2008). Anthocyanin inhibits propidium iodide DNA fluorescence in *Euphorbia pulcherrima*: implications for genome size variation and flow cytometry. *Ann. Bot.* 101 777–790. 10.1093/aob/mcm303 18158306 PMC2710214

[B9] Blanc G. Wolfe K. H. (2004). Widespread paleopolyploidy in model plant species inferred from age distributions of duplicate genes. *Plant Cell* 16 1667–1678. 10.1105/tpc.021345 15208399 PMC514152

[B10] Bures P. Wang Y. F. Horova L. Suda J. (2004). Genome size variation in Central European species of Cirsium (Compositae) and their natural hybrids. *Ann. Bot.* 94 353–363. 10.1093/aob/mch151 15292040 PMC4242176

[B11] Cesar P. M. Harumi K. Elizabeth D. E. (1994). Nuclear DNA content of ten rice species as determined by flow cytometry. *Jpn. J. Genet.* 69 513–523. 10.1266/jjg.69.513

[B12] Chen J. Huang Q. Gao D. Wang J. Lang Y. Liu T. (2013). Whole-genome sequencing of *Oryza brachyantha* reveals mechanisms underlying Oryza genome evolution. *Nat. Commun.* 4:1595. 10.1038/ncomms2596 23481403 PMC3615480

[B13] Chen S. Zhou Y. Chen Y. Gu J. (2018). Fastp: an ultra-fast all-in-one FASTQ preprocessor. *Bioinformatics* 34 i884–i890. 10.1093/bioinformatics/bty560 30423086 PMC6129281

[B14] Chen W. Hasegawa D. K. Arumuganathan K. Simmons A. M. Wintermantel W. M. Fei Z. (2015). Estimation of the whitefly *Bemisia tabaci* genome size based on k-mer and flow cytometric analyses. *Insects* 6 704–715. 10.3390/insects6030704 26463411 PMC4598660

[B15] Choi J. Y. Lye Z. N. Groen S. C. Dai X. Rughani P. Zaaijer S. (2020). Nanopore sequencing-based genome assembly and evolutionary genomics of circum-basmati rice. *Genome Biol.* 21:21. 10.1186/s13059-020-1938-2 32019604 PMC7001208

[B16] Copetti D. Zhang J. El Baidouri M. Gao D. Wang J. Barghini E. (2015). RiTE database: a resource database for genus-wide rice genomics and evolutionary biology. *BMC Genomics* 16:538. 10.1186/s12864-015-1762-3 26194356 PMC4508813

[B17] Cousin A. Heel K. Cowling W. A. Nelson M. N. (2009). An efficient high-throughput flow cytometric method for estimating DNA ploidy level in plants. *Cytom. Part A* 75 1015–1019. 10.1002/cyto.a.20816 19845019

[B18] Dodsworth S. Leitch A. R. Leitch I. J. (2015). Genome size diversity in angiosperms and its influence on gene space. *Curr. Opin. Genet. Dev.* 35 73–78. 10.1016/j.gde.2015.10.006 26605684

[B19] Dolezel J. Bartos J. (2005). Plant DNA flow cytometry and estimation of nuclear genome size. *Ann. Bot.* 95 99–110. 10.1093/aob/mci005 15596459 PMC4246710

[B20] Dolezel J. Bartos J. Voglmayr H. Greilhuber J. (2003). Nuclear DNA content and genome size of trout and human. *Cytom. Part A* 51:127-8; author reply 129. 10.1002/cyto.a.10013. 12541287

[B21] Doyle J. J. Flagel L. E. Paterson A. H. Rapp R. A. Soltis D. E. Soltis P. S. (2008). Evolutionary genetics of genome merger and doubling in plants. *Annu. Rev. Genet.* 42 443–461. 10.1146/annurev.genet.42.110807.091524 18983261

[B22] Du H. Yu Y. Ma Y. Gao Q. Cao Y. Chen Z. (2017). Sequencing and de novo assembly of a near complete indica rice genome. *Nat. Commun.* 8:15324. 10.1038/ncomms15324 28469237 PMC5418594

[B23] Flynn J. M. Hubley R. Goubert C. Rosen J. Clark A. G. Feschotte C. (2020). RepeatModeler2 for automated genomic discovery of transposable element families. *Proc. Natl. Acad. Sci. U S A.* 117 9451–9457. 10.1073/pnas.1921046117 32300014 PMC7196820

[B24] Galbraith D. W. Lambert G. M. Macas J. Dolezel J. (1997). Analysis of nuclear DNA content and ploidy in higher plants. *Curr. Protocols Cytometry* 2 6–7. 10.1002/0471142956.cy0706s02 18770733

[B25] Gao L. Z. Liu Y. L. Zhang D. Li W. Gao J. Liu Y. (2019). Evolution of *Oryza* chloroplast genomes promoted adaptation to diverse ecological habitats. *Commun. Biol.* 2:278. 10.1038/s42003-019-0531-2 31372517 PMC6659635

[B26] Gao L. McCarthy E. M. Ganko E. W. McDonald J. F. (2004). Evolutionary history of *Oryza sativa* LTR retrotransposons: a preliminary survey of the rice genome sequences. *BMC Genomics* 5:18. 10.1186/1471-2164-5-18 15040813 PMC373447

[B27] Ge S. Sang T. Lu B. R. Hong D. Y. (1999). Phylogeny of rice genomes with emphasis on origins of allotetraploid species. *Proc. Natl. Acad. Sci. U S A.* 96 14400–14405. 10.1073/pnas.96.25.14400 10588717 PMC24448

[B28] Goff S. A. Ricke D. Lan T. Presting G. Wang R. Dunn M. (2002). A draft sequence of the rice genome (*Oryza sativa* L. Ssp. Japonica). *Science* 296 92–100. 10.1126/science.1068275 11935018

[B29] Gregory T. R. (2005). The C-value enigma in plants and animals: a review of parallels and an appeal for partnership. *Ann. Bot.* 95 133–146. 10.1093/aob/mci009 15596463 PMC4246714

[B30] Greilhuber J. Dolezel J. Lysak M. A. Bennett M. D. (2005). The origin, evolution and proposed stabilization of the terms ‘genome size’ and ‘C-value’ to describe nuclear DNA contents. *Ann. Bot.* 95 255–260. 10.1093/aob/mci019 15596473 PMC4246724

[B31] Guo L. T. Wang S. L. Wu Q. J. Zhou X. G. Xie W. Zhang Y. J. (2015). Flow cytometry and K-mer analysis estimates of the genome sizes of *Bemisia tabaci* B and Q (Hemiptera: Aleyrodidae). *Front. Physiol.* 6:144. 10.3389/fphys.2015.00144 26042041 PMC4436570

[B32] Hanson L. Boyd A. Johnson M. A. Bennett M. D. (2005). First nuclear DNA C-values for 18 eudicot families. *Ann. Bot.* 96 1315–1320. 10.1093/aob/mci283 16239248 PMC4247082

[B33] Havecker E. R. Gao X. Voytas D. F. (2004). The diversity of LTR retrotransposons. *Genome Biol.* 5:225. 10.1186/gb-2004-5-6-225 15186483 PMC463057

[B34] He K. Lin K. Wang G. Li F. (2016). Genome sizes of nine insect species determined by flow cytometry and k-mer analysis. *Front. Physiol.* 7:569. 10.3389/fphys.2016.00569 27932995 PMC5121235

[B35] Huang H. Tong Y. Zhang Q. J. Gao L. Z. (2013). Genome size variation among and within *Camellia species* by using flow cytometric analysis. *PLoS One* 8:e64981. 10.1371/journal.pone.0064981 23724111 PMC3664571

[B36] Huang X. Kurata N. Wei X. Wang Z. Wang A. Zhao Q. (2012). A map of rice genome variation reveals the origin of cultivated rice. *Nature* 490 497–501. 10.1038/nature11532 23034647 PMC7518720

[B37] Isidre H. Xavier S. Salvador N. (2019). Nuclei release methods comparison for fresh leaves of rice (*Oryza sativa*) for efficient high throughput flow cytometry ploidy studies. *J. Plant Sci.* 8:31. 10.5539/jps.v8n2p31

[B38] Jain R. Jenkins J. Shu S. Chern M. Martin J. A. Copetti D. (2019). Genome sequence of the model rice variety KitaakeX. *BMC Genomics* 20:905. 10.1186/s12864-019-6262-4 31775618 PMC6882167

[B39] Letunic I. Bork P. (2021). Interactive tree of Life (iTOL) v5: an online tool for phylogenetic tree display and annotation. *Nucleic Acids Res.* 49 W293–W296. 10.1093/nar/gkab301 33885785 PMC8265157

[B40] Li C. B. Zhang D. M. Ge S. Lu B. R. Hong D. Y. (2001). Identification of genome constitution of *Oryza malampuzhaensis*. *O. Minuta*, and *O. Punctata* by multicolor genomic in situ hybridization. *Theor. Appl. Genet.* 103 204–211. 10.1007/s001220100563

[B41] Li H. Durbin R. (2010). Fast and accurate long-read alignment with Burrows-Wheeler transform. *Bioinformatics* 26 589–595. 10.1093/bioinformatics/btp698 20080505 PMC2828108

[B42] Li K. Jiang W. Hui Y. Kong M. Feng L. Y. Gao L. Z. (2021). Gapless indica rice genome reveals synergistic contributions of active transposable elements and segmental duplications to rice genome evolution. *Mol. Plant* 14 1745–1756. 10.1016/j.molp.2021.06.017 34171481

[B43] Li W. Li K. Huang Y. Shi C. Hu W. S. Zhang Y. (2020a). SMRT sequencing of the *Oryza rufipogon* genome reveals the genomic basis of rice adaptation. *Commun. Biol.* 3:167. 10.1038/s42003-020-0890-8 32265482 PMC7138787

[B44] Li W. Li K. Zhang Q. J. Zhu T. Zhang Y. Shi C. (2020b). Improved hybrid de novo genome assembly and annotation of African wild rice, *Oryza longistaminata*, from Illumina and PacBio sequencing reads. *Plant Genome* 13:e20001. 10.1002/tpg2.20001 33016624 PMC12807249

[B45] Li W. Zhang Q. J. Zhu T. Tong Y. Li K. Shi C. (2020c). Draft genomes of two outcrossing wild rice, *Oryza rufipogon* and *O. Longistaminata*, reveal genomic features associated with mating-system evolution. *Plant Direct* 4:e232. 10.1002/pld3.232 32537559 PMC7287411

[B46] Li X. Waterman M. S. (2003). Estimating the repeat structure and length of DNA sequences using L-tuples. *Genome Res.* 13 1916–1922. 10.1101/gr.1251803 12902383 PMC403783

[B47] Liu B. Shi Y. Yuan J. Hu X. Zhang H. Li N. (2013). Estimation of genomic characteristics by analyzing k-mer frequency in de novo genome projects. *ArXiv [preprint].* https://arxiv.org/abs/1308.2012 (accessed March 6, 2021).

[B48] Ma J. Devos K. M. Bennetzen J. L. (2004). Analyses of LTR-retrotransposon structures reveal recent and rapid genomic DNA loss in rice. *Genome Res.* 14 860–869. 10.1101/gr.1466204 15078861 PMC479113

[B49] McCarthy E. M. Liu J. Lizhi G. Mcdonald J. F. (2002). Long terminal repeat retrotransposons of *Oryza sativa*. *Genome Biol.* 3:H53. 10.1186/gb-2002-3-10-research0053 12372141 PMC134482

[B50] McKenna A. Hanna M. Banks E. Sivachenko A. Cibulskis K. Kernytsky A. (2010). The genome analysis toolkit: a mapreduce framework for analyzing next-generation DNA sequencing data. *Genome Res.* 20 1297–1303. 10.1101/gr.107524.110 20644199 PMC2928508

[B51] Mgwatyu Y. Stander A. A. Ferreira S. Williams W. Hesse U. (2020). Rooibos (*Aspalathus linearis*) genome size estimation using flow cytometry and K-Mer analyses. *Plants (Basel)* 9:270. 10.3390/plants9020270 32085566 PMC7076435

[B52] Miyabayashi T. Nonomura K. Morishima H. Kurata N. (2007). Genome size of twenty wild species of oryza determined by flow cytometric and chromosome analyses. *Breeding Sci.* 57 73–78. 10.1270/jsbbs.57.73 26081539

[B53] Mondal T. K. Rawal H. C. Chowrasia S. Varshney D. Panda A. K. Mazumdar A. (2018). Draft genome sequence of first monocot-halophytic species *Oryza* coarctata reveals stress-specific genes. *Sci. Rep.* 8 13613–13698. 10.1038/s41598-018-31518-y 30209320 PMC6135824

[B54] Mondal T. K. Rawal H. C. Gaikwad K. Sharma T. R. Singh N. K. (2017). First de novo draft genome sequence of *Oryza coarctata*, the only halophytic species in the genus *Oryza*. *F1000Res* 6:1750. 10.12688/f1000research.12414.2 29123646 PMC5657017

[B55] Ou S. Jiang N. (2018). LTR_retriever: a highly accurate and sensitive program for identification of long terminal repeat retrotransposons. *Plant Physiol.* 176 1410–1422. 10.1104/pp.17.01310 29233850 PMC5813529

[B56] Pflug J. M. Holmes V. R. Burrus C. Johnston J. S. Maddison D. R. (2020). Measuring genome sizes using Read-Depth, k-mers, and flow cytometry: methodological comparisons in beetles (Coleoptera). *G3-Genes Genom. Genet.* 10 3047–3060. 10.1534/g3.120.401028 32601059 PMC7466995

[B57] Piégu B. Arensburger P. Beauclair L. Chabault M. Raynaud E. Coustham V. (2020). Variations in genome size between wild and domesticated lineages of fowls belonging to the *Gallus gallus* species. *Genomics* 112 1660–1673. 10.1016/j.ygeno.2019.10.004 31669705

[B58] Piegu B. Guyot R. Picault N. Roulin A. Saniyal A. Kim H. (2006). Doubling genome size without polyploidization: dynamics of retrotransposition-driven genomic expansions in *Oryza australiensis*, a wild relative of rice. *Genome Res.* 16 1262–1269. 10.1101/gr.5290206 16963705 PMC1581435

[B59] Porebski S. Bailey L. G. Baum B. R. (1997). Modification of a CTAB DNA extraction protocol for plants containing high polysaccharide and polyphenol components. *Plant Mol. Biol. Rep.* 15 8–15. 10.1007/BF02772108

[B60] Price H. J. Hodnett G. Johnston J. S. (2000). Sunflower (*Helianthus annuus*) leaves contain compounds that reduce nuclear propidium iodide fluorescence. *Ann. Bot.* 86 929–934.

[B61] Reuscher S. Furuta T. Bessho-Uehara K. Cosi M. Jena K. K. Toyoda A. (2018). Assembling the genome of the African wild rice *Oryza longistaminata* by exploiting synteny in closely related *Oryza species*. *Commun. Biol.* 1:162. 10.1038/s42003-018-0171-y 30320230 PMC6173730

[B62] Roberts A. V. (2007). The use of bead beating to prepare suspensions of nuclei for flow cytometry from fresh leaves, herbarium leaves, petals and pollen. *Cytom. Part A* 71 1039–1044. 10.1002/cyto.a.20486 17990323

[B63] SanMiguel P. Gaut B. S. Tikhonov A. Nakajima Y. Bennetzen J. L. (1998). The paleontology of intergene retrotransposons of maize. *Nat. Genet.* 20 43–45. 10.1038/1695 9731528

[B64] Sano Y. (1980). Adaptive strategies compared between the diploid and tetraploid forms of *Oryza punctata*. *Bot. Mag.* 93 171–180. 10.1007/BF02489319

[B65] Sasaki T. (2005). The map-based sequence of the rice genome. *Nature* 436 793–800. 10.1038/nature03895 16100779

[B66] Shenton M. Kobayashi M. Terashima S. Ohyanagi H. Copetti D. Hernandez-Hernandez T. (2020). Evolution and diversity of the wild rice *Oryza officinalis* complex, across continents, genome types, and ploidy levels. *Genome Biol. Evol.* 12 413–428. 10.1093/gbe/evaa037 32125373 PMC7531200

[B67] Shi C. Li W. Zhang Q. J. Zhang Y. Tong Y. Li K. (2020). The draft genome sequence of an upland wild rice species, *Oryza granulata*. *Sci. Data* 7:131. 10.1038/s41597-020-0470-2 32350267 PMC7190833

[B68] Soltis D. E. Soltis P. S. Bennett M. D. Leitch I. J. (2003). Evolution of genome size in the angiosperms. *Am. J. Bot.* 90 1596–1603. 10.3732/ajb.90.11.1596 21653334

[B69] Soltis D. E. Visger C. J. Soltis P. S. (2014). The polyploidy revolution then.and now: stebbins revisited. *Am. J. Bot.* 101 1057–1078. 10.3732/ajb.1400178 25049267

[B70] Song J. Xie W. Wang S. Guo Y. Koo D. Kudrna D. (2021). Two gap-free reference genomes and a global view of the centromere architecture in rice. *Mol. Plant* 14 1757–1767. 10.1016/j.molp.2021.06.018 34171480

[B71] Stamatakis A. (2014). RAxML version 8: a tool for phylogenetic analysis and post-analysis of large phylogenies. *Bioinformatics* 30 1312–1313. 10.1093/bioinformatics/btu033 24451623 PMC3998144

[B72] Stein J. C. Yu Y. Copetti D. Zwickl D. J. Zhang L. Zhang C. (2018). Genomes of 13 domesticated and wild rice relatives highlight genetic conservation, turnover and innovation across the genus *Oryza*. *Nat. Genet.* 50 285–296. 10.1038/s41588-018-0040-0 29358651

[B73] Suh A. (2019). Genome size evolution: small transposons with large consequences. *Curr. Biol.* 29 R241–R243. 10.1016/j.cub.2019.02.032 30939304

[B74] Sun H. Ding J. Piednoel M. Schneeberger K. (2018). FindGSE: estimating genome size variation within human and *Arabidopsis* using k-mer frequencies. *Bioinformatics* 34 550–557. 10.1093/bioinformatics/btx637 29444236

[B75] Swift H. (1950). The constancy of desoxyribose nucleic acid in plant nuclei. *Proc. Natl. Acad. Sci. U S A.* 36 643–654. 10.1073/pnas.36.11.643 14808154 PMC1063260

[B76] Tamura K. Stecher G. Kumar S. (2021). MEGA11: molecular evolutionary genetics analysis version 11. *Mol. Biol. Evol.* 38 3022–3027. 10.1093/molbev/msab120 33892491 PMC8233496

[B77] Tanaka T. Nishijima R. Teramoto S. Kitomi Y. Hayashi T. Uga Y. (2020). De novo genome assembly of the indica rice variety IR64 using linked-read sequencing and nanopore sequencing. *G3-Genes Genom. Genet.* 10 1495–1501. 10.1534/g3.119.400871 32184372 PMC7202035

[B78] Tarailo-Graovac M. Chen N. (2009). Using repeatmasker to identify repetitive elements in genomic sequences. *Curr. Protocols Bioinform. Chapter* 4 4–10. 10.1002/0471250953.bi0410s25 19274634

[B79] Tian Z. Rizzon C. Du J. Zhu L. Bennetzen J. L. Jackson S. A. (2009). Do genetic recombination and gene density shape the pattern of DNA elimination in rice long terminal repeat retrotransposons? *Genome Res.* 19 2221–2230. 10.1101/gr.083899.108 19789376 PMC2792168

[B80] Tsutsui N. D. Suarez A. V. Spagna J. C. Johnston J. S. (2008). The evolution of genome size in ants. *BMC. Evol. Biol.* 8:64. 10.1186/1471-2148-8-64 18302783 PMC2268675

[B81] Tyagi A. Sandhya, Sharma P. Saxena S. Sharma R. Amitha M. S. (2019). The genome size of clusterbean (Cyamopsis tetragonoloba) is significantly smaller compared to its wild relatives as estimated by flow cytometry. *Gene* 707 205–211. 10.1016/j.gene.2019.02.090 30898697

[B82] Uozu S. Ikehashi H. Ohmido N. Ohtsubo H. Ohtsubo E. Fukui K. (1997). Repetitive sequences: cause for variation in genome size and chromosome morphology in the genus *Oryza*. *Plant Mol. Biol.* 35 791–799. 10.1023/A:1005823124989 9426599

[B83] Vaughan D. A. (1989). *The Genus Oryza L. Current Status of Taxonomy.* Los Banos: International Rice Research Institute. IRRI Research Paper Series.

[B84] Vitte C. Panaud O. Quesneville H. (2007). LTR retrotransposons in rice (*Oryza sativa*, L.): recent burst amplifications followed by rapid DNA loss. *BMC Genomics* 8:218. 10.1186/1471-2164-8-218 17617907 PMC1940013

[B85] Wang M. Yu Y. Haberer G. Marri P. R. Fan C. Goicoechea J. L. (2014). The genome sequence of African rice (*Oryza glaberrima*) and evidence for independent domestication. *Nat. Genet.* 46 982–988. 10.1038/ng.3044 25064006 PMC7036042

[B86] Wang X. Shi X. Hao B. Ge S. Luo J. (2005). Duplication and DNA segmental loss in the rice genome: implications for diploidization. *New Phytol.* 165 937–946. 10.1111/j.1469-8137.2004.01293.x 15720704

[B87] Wicker T. Keller B. (2007). Genome-wide comparative analysis of copia retrotransposons in Triticeae, rice, and *Arabidopsis* reveals conserved ancient evolutionary lineages and distinct dynamics of individual copia families. *Genome Res.* 17 1072–1081. 10.1101/gr.6214107 17556529 PMC1899118

[B88] Wicker T. Sabot F. Hua-Van A. Bennetzen J. L. Capy P. Chalhoub B. (2007). A unified classification system for eukaryotic transposable elements. *Nat. Rev. Genet.* 8 973–982. 10.1038/nrg2165 17984973

[B89] Wing R. A. Purugganan M. D. Zhang Q. (2018). The rice genome revolution: from an ancient grain to Green Super Rice. *Nat. Rev. Genet.* 19 505–517. 10.1038/s41576-018-0024-z 29872215

[B90] Wu Z. Fang D. Yang R. Gao F. An X. Zhuo X. (2018). De novo genome assembly of *Oryza granulata* reveals rapid genome expansion and adaptive evolution. *Commun. Biol.* 1:84. 10.1038/s42003-018-0089-4 30271965 PMC6123737

[B91] Xie X. Du H. Tang H. Tang J. Tan X. Liu W. (2021). A chromosome-level genome assembly of the wild rice *Oryza rufipogon* facilitates tracing the origins of Asian cultivated rice. *Sci. China Life Sci.* 64 282–293. 10.1007/s11427-020-1738-x 32737856

[B92] Xu Z. Wang H. (2007). LTR_FINDER: an efficient tool for the prediction of full-length LTR retrotransposons. *Nucleic Acids Res.* 35 W265–W268. 10.1093/nar/gkm286 17485477 PMC1933203

[B93] Yu H. Lin T. Meng X. Du H. Zhang J. Liu G. (2021). A route to de novo domestication of wild allotetraploid rice. *Cell* 184 1156–1170. 10.1016/j.cell.2021.01.013 33539781

[B94] Yu J. Hu S. Wang J. Wong G. K. Li S. Liu B. (2002). A draft sequence of the rice genome (*Oryza sativa* L. Ssp. Indica). *Science* 296 79–92. 10.1126/science.1068037 11935017

[B95] Zhang J. Chen L. Xing F. Kudrna D. A. Yao W. Copetti D. (2016). Extensive sequence divergence between the reference genomes of two elite indica rice varieties Zhenshan 97 and Minghui 63. *Proc. Natl. Acad. Sci. U S A.* 113 E5163–E5171. 10.1073/pnas.1611012113 27535938 PMC5024649

[B96] Zhang Q. J. Gao L. Z. (2017). Rapid and recent evolution of LTR retrotransposons drives rice genome evolution during the speciation of AA-Genome *Oryza species*. *G3-Genes Genom. Genet.* 7 1875–1885. 10.1534/g3.116.037572 28413161 PMC5473765

[B97] Zhang Q. J. Zhu T. Xia E. H. Shi C. Liu Y. L. Zhang Y. (2014). Rapid diversification of five *Oryza* AA genomes associated with rice adaptation. *Proc. Natl. Acad. Sci. U S A.* 111 E4954–E4962. 10.1073/pnas.1418307111 25368197 PMC4246335

[B98] Zhang Q. Liang Z. Cui X. Ji C. Li Y. Zhang P. (2018). N(6)-Methyladenine DNA methylation in japonica and indica rice genomes and its association with gene expression, plant development, and stress responses. *Mol. Plant* 11 1492–1508. 10.1016/j.molp.2018.11.005 30448535

[B99] Zhang R. G. Li G. Y. Wang X. L. Dainat J. Wang Z. X. Ou S. (2022). TEsorter: an accurate and fast method to classify LTR-retrotransposons in plant genomes. *Hortic. Res.* 9:c17. 10.1093/hr/uhac017 35184178 PMC9002660

[B100] Zhang Y. Zhang S. Zhang J. Liu H. Fu B. Li X. (2015). Genome and comparative transcriptomics of african wild rice *Oryza longistaminata* provide insights into molecular mechanism of rhizomatousness and self-incompatibility. *Mol. Plant* 8 1683–1686. 10.1016/j.molp.2015.08.006 26358679

[B101] Zhao Q. Feng Q. Lu H. Li Y. Wang A. Tian Q. (2018). Pan-genome analysis highlights the extent of genomic variation in cultivated and wild rice. *Nat. Genet.* 50 278–284. 10.1038/s41588-018-0041-z 29335547

[B102] Zhou S. Yan X. Zhang K. Liu H. Xu J. Nie S. (2021). A comprehensive annotation dataset of intact LTR retrotransposons of 300 plant genomes. *Sci. Data* 8:174. 10.1038/s41597-021-00968-x 34267227 PMC8282616

[B103] Zou X. H. Du Y. S. Tang L. Xu X. W. Doyle J. J. Sang T. (2015). Multiple origins of BBCC allopolyploid species in the rice genus (*Oryza*). *Sci. Rep.* 5:14876. 10.1038/srep14876 26460928 PMC4602239

[B104] Zuccolo A. Sebastian A. Talag J. Yu Y. Kim H. Collura K. (2007). Transposable element distribution, abundance and role in genome size variation in the genus *Oryza*. *BMC Evol. Biol.* 7:152. 10.1186/1471-2148-7-152 17727727 PMC2041954

